# Exploring the relationship between metacognitive awareness and Chinese EFL learners’ listening skills

**DOI:** 10.3389/fpsyg.2023.1148610

**Published:** 2023-05-02

**Authors:** Ying Fu, Min Wang, Shangchao Min, Songbo Zhou, Xunyi Pan

**Affiliations:** School of International Studies, Zhejiang University, Hangzhou, China

**Keywords:** metacognitive awareness, the MALQ, cognitive diagnostic assessment, listening subskills, EFL learners

## Abstract

Listening causes great difficulties for EFL learners and little is known concerning the contribution of EFL learners’ metacognitive awareness to their listening performance and to their mastery of listening subskill. In the present study, the Metacognitive Awareness Listening Questionnaire (MALQ) and an in-house listening test were used to collect data from 567 Chinese EFL college students. The G-DINA package in R was adopted to identify students’ mastery patterns of listening subskills. The correlations of test takers’ MALQ results and their listening scores and listening subskills mastery probability were analyzed, respectively, to investigate how test participants’ metacognitive awareness relates to their language proficiency and listening subskills. According to the study, learners’ metacognitive awareness has a significant positive relationship with their listening performance at overall and subskills levels. The findings of the study provide additional evidence for using the MALQ as an instrument to interpret learners’ metacognitive awareness of listening strategies. It is thus recommended that theorists and language teachers involve metacognitive awareness of strategies in listening instructions.

## 1. Introduction

Listening causes great difficulties for second language (L2) learners because of the complexity, the unidirectionality and the intangibility of listening comprehension process (Goh, 2000; Hasan, 2000; Graham, 2006, 2011; Vandergrift, 2007; Prince, 2013). Arguably, it is “a source of frustration to learners” (Graham, 2011, p. 113). In the same vein, listening is the least researched compared with the other three language skills, namely, speaking, reading and writing, given that it is an implicit process difficult to access (Vandergrift, 2007). Therefore, what factors contribute to the development of L2 listening is an issue of great theoretical and pedagogical implications. One factor that has been widely investigated in the past decade is metacognition, which is an umbrella term for learners’ knowledge about and regulation of their cognitive activities in the learning process (Flavell, 1979; Zeng and Goh, 2015). Previous research on the role of metacognition in L2 listening mainly focuses on metacognitive awareness by defining metacognition as “listener awareness of the cognitive processes involved in comprehension and the capacity to oversee, regulate and direct these processes (Vandergrift and Baker, 2018, p. 85).” There is evidence that metacognitive awareness and strategies might have positive impact on L2 learners’ performance in listening proficiency tests (e.g., Victori and Lockhart, 1995; Bolitho et al., 2003; Wilson, 2003; Vandergrift and Baker, 2015; Zeng and Goh, 2015). However, the relationship between these two factors might not be simple or straightforward in that the findings of existing studies are mixed and inconclusive (e.g., Goh and Hu, 2013; Zuo, 2013; Vandergrift and Baker, 2015, 2018; Zeng and Goh, 2015; Wang and Treffers-Daller, 2017; Wallace, 2020). While some researchers observed a direct and significant relationship between the two factors (e.g., Vandergrift and Baker, 2015; Zeng and Goh, 2015; Xu and Huang, 2018; Du and Man, 2022), others claimed that metacognitive awareness does not have a direct contribution to listening comprehension performance (e.g., Wang and Treffers-Daller, 2017; Wallace, 2020).

Another issue that remains unclear in previous research is how metacognitive awareness is correlated with subskills of L2 listening. More specifically, only overall listening performance is considered when investigating the relationship between metacognition and L2 listening, leaving it unanswered whether metacognitive awareness modulates the development of different listening subskills (attribute) in the same fashion. This gap renders our incomplete understanding on how metacognition is related to L2 listening, thereby undermining the theoretical and pedagogical implications of the research in this respect. To address this gap, this study employs a cognitive diagnostic model (G-DINA) to classify EFL (English as a Foreign Language) learners’ listening subskills and probe into the relationship between the components of metacognitive awareness and specific listening subskills. Hopefully, the findings of the present study can shed light on what role metacognitive awareness of strategies plays in listening comprehension process, providing insights into L2 instruction of listening comprehension.

## 2. Literature review

### 2.1. Metacognitive awareness and listening proficiency

Metacognition refers to both knowledge concerning one’s cognitive processes and the capacity to regulate and control these processes (Flavell, 1976). Based on this concept, Vandergrift and Goh (2012) defined metacognition in listening as the “listeners’ awareness of their cognitive processes (p. 395)” in listening comprehension and their capacity to monitor and regulate the processes. The term “listening metacognitive awareness” was thus initiated, which is argued to consist of three components namely, metacognitive experience (i.e., how listeners feel about their learning), metacognitive knowledge (i.e., what knowledge listeners have in their mind about listening), and strategy use (i.e., the techniques listeners adopt to help their listening). Inspired by this three-component framework, many researchers conducted research to explore the relationship between metacognition and listening proficiency with a focus on L2 listeners’ use of listening comprehension strategies (Goh, 2002; Vandergrift, 2003a; Smidt and Hegelheimer, 2004). With the dataset based on a retrospective verbalization procedure, Goh (2002) investigated the listening strategies employed by Chinese learners of English and found that those with higher listening proficiency used metacognitive strategies more effectively than those with lower proficiency. By recording, transcribing, and analyzing the think-aloud protocols, Vandergrift (2003a) found skilled listeners adopted much more metacognitive strategies compared with their less-skilled counterparts. Notably, the two groups of learners differed greatly in the type of strategies they used: the skilled group primarily used comprehension monitoring, while less-skilled listeners used much more on-line translation.

Drawing on implications from the research above, Vandergrift et al. (2006) developed and validated a five-factor model, the Metacognitive Awareness Listening Questionnaire (MALQ), “to assess L2 learners’ metacognitive awareness and perceived use of listening strategies (p. 431).” Five strategic factors concerning metacognitive awareness were identified including “*Problem-solving*, *Planning and Evaluation*, *Mental Translation*, *Person Knowledge and Directed Attention* (Vandergrift et al., 2006, p. 449).” In the process of validating the MALQ, they observed a significant relationship between metacognition and listening comprehension success. More specifically, they found that metacognition could explain “about 13% of the variance in listening performance (p. 449).”

Hitherto MALQ has been extensively adopted to study the impact of metacognition on listening comprehension (e.g., Goh and Hu, 2013; Zuo, 2013; Vandergrift and Baker, 2015; Zeng and Goh, 2015; Shen and Zhang, 2016; Xu and Huang, 2018; Du and Man, 2022). Goh and Hu (2013) examined how metacognitive awareness exerted impact on ESL learners’ listening proficiency measured by the IELTS sample test. It turned out that MALQ scores could explain 22% of the variances in listening test scores for intermediate-level L2 students in Singapore. By using exploratory path analysis, Vandergrift and Baker (2015) revealed a positive correlation between listening metacognitive awareness and students’ test performance. In the same vein, Zeng and Goh (2015) showed that MALQ scores could explain 13–15% of the variances in low-level Chinese ESL learners’ standardized listening test scores. In addition to the direct impact on listening performance, metacognitive awareness has been found to modulate the relationships between test performance and other factors of individual differences. For instance, Xu and Huang (2018) demonstrated that test anxiety affected L2 listening test performance and the effect was mediated by metacognitive awareness. Du and Man (2022) investigated L2 students’ person factors and strategic processing during the listening process and found that listening metacognitive knowledge is significantly correlated with L2 listening performance.

More recent research revealed an indirect and non-linear relationship between metacognition and listening comprehension. Wang and Treffers-Daller (2017), for example, showed that metacognitive awareness may not be directly related to listening comprehension as MALQ scores did not contribute significantly to listening comprehension performance, with an exception of person knowledge, which demonstrated a significant correlation with L2 listening skills. In addition, Vandergrift and Baker (2018) discovered that metacognitive awareness was not a significant predictor of L2 listening comprehension in French immersion classrooms, but their path model hinted the effect of metacognitive awareness on students’ listening performance might be mediated by their vocabulary knowledge (Wallace, 2020). Unlike Vandergrift and Baker (2015) who argued that listeners used their metacognitive awareness for compensation of unknown words, Wallace (2020) argued that metacognitive awareness had an indirect impact on listening and helped listeners deal with comprehension difficulties by using their topical knowledge. He further explained that the weak predictive power of metacognitive awareness on listening performance may indicate that it was just one of “the domain-general peripheral factors” (Wallace, 2020, p. 32) and would only indirectly affect listening comprehension through students’ knowledge of core vocabulary and specific domain topics.

Notably, the “indirect-effect-explanation” is at odds with the findings from the research where metacognition was adopted as one single factor (Goh and Hu, 2013; Zuo, 2013; Zeng and Goh, 2015; Shen and Zhang, 2016; Xu and Huang, 2018) or one of the factors (Vandergrift and Baker, 2015; Du and Man, 2022), which demonstrated that metacognition did explain a certain amount of variance in listening success. Moreover, disagreements in existing studies could be attributed to, at least partially the methodological issues. As a common, standardized way of measuring listening proficiency was yet to find (Berne, 2004), they used different listening tests to assess listening proficiency, making the results incomparable. More importantly, they primarily used overall test scores to measure listening proficiency. As a result, it remains unclear how metacognitive awareness affects listening subskills. Addressing this issue, however, has great theoretical and pedagogical implications. On one hand, it will deepen our understanding of the mechanism underlying the relationship between metacognition and listening comprehension. On the other hand, it will inform L2 teaching of listening comprehension by specifying what metacognitive strategies are more facilitative to the development of listening skills. Taken together, more research is needed to further study the relationship between metacognitive awareness and L2 listening proficiency. To this end, the present research employed MCQ and the cognitive diagnosis approach (CDA) to investigate how metacognitive awareness is correlated with L2 learners’ listening subskills as well as overall listening proficiency.

### 2.2. Cognitive diagnosis in L2 listening assessments

Cognitive diagnosis aims to identify learner strengths and weaknesses by extracting fine-grained information from test performance data (Lee and Sawaki, 2009; Zhan and Qiao, 2022). Underpinned by theories of cognitive psychology and psychometric modeling, cognitive diagnosis approaches (CDAs) make it possible to assess L2 learners’ state of knowledge and skill mastery simultaneously. CDAs involve (1) analyzing the test items and identifying cognitive skills involved, and (2) mathematical modeling of test takers’ skill mastery patterns according to their responses to the test and the identified cognitive skills. Recent years have witnessed the development of a variety of cognitive diagnosis models (CDMs) and their application to educational assessment (e.g., Liu et al., 2018; Aryadoust, 2021; He et al., 2022; Min et al., 2022a,b), including the rule space model, the tree-based regression, the FUSION Model, G-DINA, ACDM, C-RUM, DINO, DINA, RRUM, G-DINA, the deep CDM, the sequential hierarchical CDM, and the semi-supervised learning ANN for diagnostic classification (Zhang and Wang, 2020; Xue and Bradshaw, 2021; Gao et al., 2022).

Cognitive diagnosis models can be categorized as compensatory, non-compensatory and saturated models. Compensatory models posit that non-mastery of one attribute can be compensated for by the mastery of another attribute. The ACDM (de la Torre, 2011) and the DINO (Templin and Henson, 2006) are two typical examples of compensatory models, with the DINO model providing the most extreme scenario of all compensations, in which any attribute can totally compensate for the absence of all the other attributes (Yi, 2017). Non-compensatory models assume that missing an attribute is the same as missing all required attributes. Typical examples are the RRUM (DiBello et al., 2007) and the DINA (Junker and Sijtsma, 2001). The DINA model is the most restrictive non-compensatory model in that candidates can answer the item correctly only if they have mastered all the attributes measured by the item (Liu et al., 2018). Saturated models are more flexible than the other two types of CDMS when representing the relationship among different attributes and can be used even when the relationship among attributes are unknown (Li et al., 2016). The generalized DINA model, or the G-DINA, is a commonly used example (de la Torre, 2011). As is suggested in its name, G-DINA is a general model that includes many CDA models including the models mentioned above.

Non-compensatory CDAs, such as the rule space model (Tatsuoka, 1990) and the tree based regression (Sheehan, 1997), were adopted in early listening assessment research for creating and evaluating a Q-matrix, which characterizes the relationship between test items and subskills. Other models are applied to analyze test response data associated with a given Q-matrix (e.g., Lee and Sawaki, 2009; Aryadoust, 2011; Yi, 2017; Min et al., 2022a; He et al., 2022) and examine skill-mastery classification results.

Cognitive diagnosis approaches to listening comprehension assessment are comparatively under-represented because of the “Cinderella” status of listening among the four L2 learning skills (Harding et al., 2015). Still, researchers of listening assessment agree on the multidimensional and divisible trait of listening comprehension (Buck and Tatsuoka, 1998; Song, 2008; Aryadoust, 2021). Various listening subskills have been identified, “ranging from the simplest dichotomies to very detailed lists” (Buck and Tatsuoka, 1998, p. 121) even though a consensus on taxonomies for listening skills is yet to reach among researchers (e.g., Buck, 2001; Harding et al., 2015).

Buck and Tatsuoka (1998) adopted the rule-space model to explore the cognitive and linguistic attributes underlying the listening comprehension performance and identified 15 cognitive attributes and 14 interactions in the final attribute list including both task and text features and test taker ability. Sawaki et al. (2009) used the fusion model and identified four meaningful subskills examined in the Reading and Listening sections of TOEFL test (Test of English as a Foreign Language). Lee and Sawaki (2009) applied three CDAs to the response data from TOEFL iBT listening test. Four identical listening skills are identified in both studies (Lee and Sawaki, 2009; Sawaki et al., 2009) involving the understanding of general and specific information, the understanding of text structure and speaker intention, and skills to connect ideas. Aryadoust (2011) applied the fusion model to a while-listening performance (WLP) test and found evidence for eight subskills, putting emphasis on paraphrasing skills, grammatical forms and information density.

More recently, with the application of five CDAs, Aryadoust (2021) identified nine listening-specific subskills and test-specific facets extracted from the response data from the listening test of the Singapore–Cambridge General Certificate of Education (GCE) exam. Apart from paraphrasing skills, he identified skills including making pragmatic or general inferences, understanding surface information and details, eliminating inaccurate information and making anaphoric moves in the text, etc., Yi (2017) tried to select the best fitting model from five cognitive diagnostic assessment models by using the response data from a 51-item mock TOEFL listening test. Three skills are identified based on the framework proposed by Douglas et al. (2000), involving not only the understanding of basic and pragmatic information, but also the understanding of organization and connecting information. Using the performance data of two cognitive diagnostic models in an in-house EFL listening test, Min et al. (2022b) identified four attributes: listening for words, details, intended meaning, and main idea. He et al. (2022) explored the similarities and differences in EFL learners’ use of local and global subskills, based on listening and reading test data from a large-scale English in-house test for students who intend to get their bachelor’s degree. The subskills identified in this study are: linguistic knowledge, details, synthesizing, and inferencing.

All the research demonstrates that the CDAs are a versatile tool of identifying subskills of L2 listening. Accordingly, using CDAs to assess listening enables us to zoom in the contribution of metacognitive awareness to subskills of L2 listening proficiency.

### 2.3. The present study

The present study explores how EFL learners’ metacognitive awareness impacts their listening comprehension performance. Following previous research (e.g., Goh and Hu, 2013; Zeng and Goh, 2015; Wang and Treffers-Daller, 2017; Xu and Huang, 2018; Wallace, 2020; Du and Man, 2022), we used MALQ to measure EFL learners’ metacognitive awareness d by MALQ. At the same time, CDA was adopted to identify the listening subskills presented in the test and analyze how these subskills are modulated by metacognitive factors.

### 2.4. Research questions

The present study addresses the following two research questions:

1) To what extent does EFL students’ metacognitive awareness relate to their overall listening performance?
2) To what extent does EFL students’ metacognitive awareness relate to their listening subskills?

## 3. Materials and methods

### 3.1. Participants

#### 3.1.1. Coders

Four female experts coded the listening subskills measured by the listening test. All the coders are faculty members of a key university in China and have experience in teaching EFL at tertiary level. One of the coders was involved in the development of the listening scale of China’s Standards of English Language Ability (the CSE) and all of them have considerable knowledge in language testing.

#### 3.1.2. Test takers

The researchers randomly selected 18 classes of non-English major freshmen at a prestigious Chinese university for the study. All the 614 students from the 18 classes were enrolled in the compulsory English courses based on the National College English Teaching Syllabus (The Ministry of Education of the People’s Republic of China, 2007). The data of 567 participants were included in the study since 47 students did not complete both the questionnaire and the listening test.

### 3.2. Instruments

#### 3.2.1. Listening comprehension test of an in-house English proficiency test

The listening comprehension test is taken from an in-house EPT, a test for all non-English majors who expect to graduate with their bachelor’s degree. The EPT is administered twice an academic year. After completing their first-year EFL courses, students can take the test from the second year for 1–6 times depending on whether they pass the exam. The test comprises four subtests, i.e., listening, reading, speaking, and writing. Each subtest is individually scored. Based on the standard setting study in Min and Jiang (2020), the passing scores of listening, reading and writing subtests were aligned to China’s Standards of English Language Ability Level 5 (CSE-5).

The current research put focus on the listening comprehension subtest, a 30 min test comprising three parts with a total of 30 items. All the items have four options and are presented in a multiple-choice format. In Part I, there are 10 short conversations, each followed by one question. Part II consists of one long talk, followed by 5 questions. In Part III, there are three short passages, each followed by 5 questions. The listening test has a maximum score of 30. The recording for the listening test is played only once. Test takers can take notes during the listening process. A variety of topics are included in the listening test, covering daily life (e.g., eating at a restaurant), career (e.g., performance evaluation), science (e.g., measuring system), and people (e.g., professional development).

In light of the view that listening is a process to use one’s cognitive processing and multiple knowledge to complete listening tasks (Vandergrift and Goh, 2012), the listening comprehension test employed in the current study is developed to measure the following listening attributes: “*(1) understanding words and syntactic structures*, *(2) extracting detailed information*, *(3) making inferences*, and *(4) recognizing the main idea or the speaker’s attitude and intention* (Min and He, 2022, p. 98).”

#### 3.2.2. Metacognitive awareness questionnaire

To assess the participants’ metacognitive awareness and their use of listening strategies, the MALQ (Vandergrift et al., 2006) was used. The MALQ is a 21-item self-assessment instrument (see Appendix Table 1), using a 6-point Likert scale ranging from “strongly agree” to “strongly disagree.” All the items on the questionnaire are randomized and categorized into five groups: “*problem-solving, planning and evaluation, person knowledge, translation, and directed attention* (Vandergrift et al., 2006, p. 449).”

Two modifications were made to make the MALQ applicable for Chinese participants. First, some of original statements were reworded. For example, the statement “I feel that listening comprehension in French is a challenge for me” was changed into “I feel that listening comprehension in English is a challenge for me.” Second, all the items in the MALQ were translated from English to Chinese.

Three items (items 3, 8, and 16) in the MALQ were reversely coded prior to data analysis to ensure that the high score of each item referred to the same type of response.

### 3.3. Procedure

Both the listening comprehension test and the MALQ were carried out under controlled conditions in regularly scheduled class sessions in November, 2021. All the test takers were asked to make the right choice and put the right answer on a separate answer sheet after they had finished listening to the oral texts and the corresponding questions once. 30 min later, when all the answer sheets were collected, a copy of the MALQ was handed out and all the test takers were requested to give their responses to it and return it in 5 min.

### 3.4. Ethical considerations

Participants were all informed about the purposes of the research and the consent was obtained from all the test takers and their teachers before the study.

### 3.5. Data analysis

Data collected from both MALQ responses and the listening test were analyzed with SPSS 23.0 for Windows to address the first research question concerning the relationship between EFL learners’ metacognitive awareness and their listening proficiency. All the quantitative data were subjected to normality tests before further investigation. Firstly, the reliability and the validity of the MALQ was investigated and a principal component analysis was carried out on the 21 items using varimax rotation. Cronbach’s alpha was then calculated for each variable that contributed to the factors identified in the principal component analysis.

The cognitive diagnostic analyses were carried out by using the “GDINA” package, version 2.7.8 in R (Ma et al., 2020). Relative fit statistics and the absolute fit statistics, were used to compare G-DINA, DINA, DINO, and ACDM models through the “GDINA” package and determine the optimal model and the fit of the models to the data. Because of the saturated characteristic of G-DINA, many CDM studies, e.g., Chen and Chen (2016), often use it to analyze the effect and the interaction of subskills in L2 listening and reading comprehension (Nassaji, 2002). It usually comes up with the best absolute fit indices as it is highly parameterized. DINA, DINO, and ACDM were used for comparison in this study as G-DINA may not produce optimal relative fit indices when the sample size is not large enough. −2 log likelihood and AIC (Akaike’s information criteria) values were used in determining the best model. Smaller relative fit statistics stand for better model-data fit.

Attribute mastery prevalence was evaluated after the best-fitting model was decided. Attribute prevalence shows the proportion of those who have the mastery of the attribute. The attribute prevalence of above 0.5 for a particular attribute indicates more than 50% of the participants have demonstrated mastery of the specific attribute. Then attribute classification results were evaluated across models. Finally, Pearson correlation coefficient was calculated to examine the correlation between the attributes identified from G-DINA and the factors of metacognitive awareness contributing to listening performance.

## 4. Results

### 4.1. Metacognitive awareness and listening performance

The factor structure of the questionnaire was examined using Exploratory Factor Analysis. According to KMO and Bartlett’s Test, we had a KMO value of 0.852 when *p* = 0.000 (*p* < 0.05) (Table 1). This indicated that the degree of information among the variables overlapped greatly and presented a strong partial correlation. Hence, it was plausible to conduct factor analysis.

**TABLE 1:** Factor loadings based on a principal component analysis with varimax rotation for 21 items from the MALQ.

Items			Factors		
	**Problem-solving**	**Directed attention**	**Mental translation**	**Person knowledge**	**Planning-evaluation**
N17	0.796				
N5	0.744				
N19	0.726				
N9	0.700				
N7	0.554				
N13	0.416				
N6		0.822			
N12		0.816			
N2		0.676			
RN16		0.617			
N4			0.867		
N18			0.815		
N11			0.671		
RN8				0.870	
RN3				0.801	
N15				0.594	
N20					0.707
N21					0.660
N14					0.628
N10					0.458
N1					0.382

Extraction method: principal component analysis. Rotation method: varimax with Kaiser normalization.

The 21 items in the MALQ were subjected to a principal component analysis with varimax rotation. Five factors in the data with eigenvalues over Kaiser’s criterion of 1 are identified through the factor analysis and they explained 58.278% of the variance in combination. Following Vandergrift et al. (2006), p. 449, we labeled the five factors as “*problem-solving, mental translation, person knowledge, directed attention and planning-evaluation*” (see Table 1).

Then, we calculated Cronbach’s alpha for each variable that contributed to the five factors (see Table 2). Three of subscales, *problem-solving*, *directed attention* and *mental translation* had high reliability (Cronbach’s alpha = 0.808, 0.768, 0.763, respectively). Also, the other two subscales, namely, *person knowledge* and p*lanning-evaluation* had an acceptable reliability with Cronbach’s alpha being 0.668 and 0.645, respectively.

**TABLE 2:** Descriptive statistics for the five MALQ factors.

Factors	Number of items	Cronbach’s alpha
Problem-solving	6 (items 5, 7, 9, 13, 17, 19)	0.808
Directed attention	4 (items 2, 6, 12, 16)	0.768
Mental translation	3 (items 4, 11, 18)	0.763
Person knowledge	3 (items 3, 8, 15)	0.668
Planning-evaluation	5 (items 1, 10, 14, 20, 21)	0.645

Table 3 showed a significant correlation, ranging from −0.157 to 0.538, among the five metacognitive factors, either positive or negative, demonstrating an interacted and differing relationship among the five factors. *Problem-solving* significantly correlates with *planning and evaluation* with *r* = 0.538 *p* < 0.01, and *Mental translation* exerts negative influence on *person knowledge* (*r* = −0.157, *p* < 0.01).

**TABLE 3:** Correlations among metacognitive factors.

	Planning-evaluation	Directed attention	Person knowledge	Mental translation	Problem-solving
Planning-evaluation	1	0.367**	0.194*	0.287**	0.538**
Directed attention		1	0.272**	0.119**	0.500*
Person knowledge			1	−0.157**	0.233**
Mental translation				1	0.283**
Problem-solving					1

**Correlation is significant at the 0.01 level (2-tailed).

*Correlation is significant at the 0.05 level (2-tailed).

To figure out the relationship between the responses to the MALQ and listening performance, we correlated the data from the MALQ with the listening test scores of the test takers. A significant positive correlation was found between the two variables (*r* = 0.205, *p* < 0.01), suggesting that *metacognitive awareness* is associated with the increase in L2 listening comprehension.

### 4.2. Metacognitive awareness and EFL listening subskills

Five commonly used CDMs were first fitted to the data and examined to determine the optimal model for further analyses, including the G-DINA, DINA, ACDM, RRUM, and DINO. Table 4 summarizes the model–data fit indices for the five models. The −2 log likelihood and AIC values suggested that the G-DINA model was the best fitting model, whereas the BIC, CAIC, and SAIBC indicated that the ACDM was the best. This is expected, as these three indices often impose more penalties for complex models (Liu et al., 2018), such as the G-DINA model. Considering that the likelihood ratio test, as shown in the last three columns of Table 4, suggested that the G-DINA fit the data significantly better than the other four models, it was therefore chosen as the final model for analysis.

**TABLE 4:** Summary of model-data fit.

Model	#par	−2LL	AIC	BIC	CAIC	SAIBC	χ^2^	df	*p*-value
G-DINA	103	47,364.51	47,570.51	48,110.3	48,213.3	47,783.11			
DINA	75	47,542.84	47,692.84	48,085.89	48,160.89	47,847.64	178.33	28	<0.001
ACDM	88	47,406.37	47,582.37	48,043.55	48,131.55	47,764.01	41.86	15	<0.001
RRUM	88	47,408.77	47,584.77	48,045.95	48,133.95	47,766.41	44.26	15	<0.001
DINO	75	47,544.06	47,694.06	48,087.11	48,162.11	47,848.87	179.55	28	<0.001

Subskills mastery information was then evaluated to determine the consistency and variability of subskills performance. Both the individual subskills mastery profiles and the whole group were examined. Because of space limitation, only the attribute mastery prevalence of the group was presented. As can be seen from Table 5, attribute 3, *making inferences* was the best-mastered (with a mastery probability of 71.00%); followed by attribute 1, *understanding words* (with a mastery probability of 63.5%) and that of attribute 2, *extracting detailed information* (with a mastery probability of 63.2%). Attribute 4 (*recognizing main ideas*) was the least mastered with a probability of 56.9%.

**TABLE 5:** Attribute prevalence and classification accuracy.

Attributes	Mastery prevalence	Attribute-level classification accuracy
A1: Understanding words and syntactic structures	0.635	0.8786
A2: Extracting detailed information	0.632	0.8817
A3: Making inferences	0.710	0.8602
A4: Recognizing main ideas	0.569	0.9067

In addition, Table 5 presents the attribute-level classification accuracy. All the statistics were above 0.85, suggesting the reliability of categorizing the participants into two groups: those who master or do not master the subskills.

### 4.3. Correlation between metacognitive factors and listening subskills

To further analyze what role metacognitive awareness plays in listening process, we calculated Pearson correlation coefficient to examine the correlation between the five metacognitive factors and the listening subskills tested in the Diagnostic English Tracking Assessment (see Table 6). As is shown in Table 6, metacognitive factors as a whole have a significant correlation with all of the listening subskills except for *understanding words and syntactic structures*. Four of the five factors of MALQ, namely *person knowledge, problem-solving, directed attention and planning-evaluation*, were significantly correlated with the listening subskills, with the correlation coefficients ranging from *r* = −0.165 to *r* = 0.348, *p* < 0.01. Among the five factors, *person knowledge* shows a significant correlation with all four listening subskills, especially with *extracting detailed information* (*r* = 0.348, *p* < 0.01). In contrast, the correlation coefficients between *planning-evaluation* with four listening subskills are rather low. Comparatively speaking, however, its correlation coefficient with *recognizing the main idea* or the speaker’s *attitude and intention* is higher than that with the other three listening subskills, with *r* = 0.082, *p* < 0.01. *Directed attention* has the highest correlation with *recognizing the main idea* or the speaker’s *attitude and intention* (*r* = 0.147, *p* < 0.01), compared with its correlation with the other three listening subskills. *Problem-solving* presents a positive correlation with all the listening subskills, especially with *making inferences* (*r* = 0.172, *p* < 0.01). The only one factor that shows a negative correlation with all listening subskills is *mental translation*, ranging from *r* = −0.165 to *r* = −0.035, *p* < 0.01.

**TABLE 6:** Correlations between metacognitive awareness and listening subskills.

		Planning- evaluation	Directed attention	Person knowledge	Mental translation	Problem-solving	Metacognitive awareness
Understanding words and syntactic structures	Pearson correlation sig. (2-tailed)	0.024 0.574	0.059 0.163	0.105* 0.012	−0.035 0.400	0.058 0.165	0.064 0.127
Extracting detailed information	Pearson correlation sig. (2-tailed) N	0.072 0.086	0.137** 0.001	0.348** 0.000	−0.165** 0.000	0.146* 0.000	0.163** 0.000
Making inferences	Pearson correlation sig. (2-tailed) N	0.067 0.112	0.132 0.002	0.301** 0.000	−0.129** 0.002	0.172** 0.000	0.167** 0.000
Recognizing main ideas	Pearson correlation sig. (2-tailed) N	0.082 0.050	0.147** 0.000	0.333** 0.000	−0.153** 0.000	0.161** 0.000	0.175** 0.000

**Correlation is significant at the 0.01 level (2-tailed).

*Correlation is significant at the 0.05 level (2-tailed).

## 5. Discussion

### 5.1. To what extent does EFL students’ metacognitive awareness of strategies relate to their listening performance?

The present study yielded two major findings: First, Chinese students’ metacognitive awareness of listening was significantly correlated with their listening performance as measured by overall test scores; second, L2 listening subskills identified by CDA were in general correlated with metacognitive awareness though the degree of correlation varied. The following discussion is centered on the two findings.

For RQ1 addressing the relationship between metacognitive awareness and L2 listening performance, a significant correlation is obtained between them. This finding is consistent with previous studies (e.g., Zuo, 2013; Vandergrift and Baker, 2015; Zeng and Goh, 2015; Xu and Huang, 2018; Du and Man, 2022), confirming again that metacognitive awareness has a role to play in L2 listening comprehension. It is thus suggested that promoting metacognitive knowledge and the use of metacognitive strategy has great potentials of facilitating L2 listening comprehension. More interestingly, we found a significant correlation among the five metacognitive factors (see Table 3), which shows that t they interact with one another but in different directions. For example, problem-solving was significantly correlated with planning and evaluation on one hand and directed attention on the other. It is thus hinted that these factors conspire or work jointly in the process of L2 listening. In contrast, mental translation is negatively correlated with person knowledge, which suggests the implementation of mental translation in L2 listening might impede the use of person knowledge, which has been proved to be beneficial to L2 listening (Wang and Treffers-Daller, 2017). This stipulation is in congruence with the view that mental translation is an inefficient approach that listeners should overcome if they intend to be skilled in L2 listening (Vandergrift et al., 2006). In fact, when Vandergrift et al. (2006) developed and validated the MALQ, he found a relatively strong interrelation among metacognitive factors and took it as “further evidence for the complexity and interconnectedness of these metacognitive processes (Vandergrift et al., 2006, p. 451),” or “orchestration,” as defined by Anderson (1999) and Vandergrift (2003a). Taken together, the interactions between the components of metacognitive awareness might lead to an offset of the contribution of metacognitive awareness to L2 listening performance. Therefore, it is difficult, if not impossible to determine whether metacognitive awareness is associated with L2 listening performance or not. Instead, more research is warranted to specify the conditions under which metacognitive awareness can or cannot assist in L2 listening comprehension. This is the reason why the present study scrutinized how metacognitive awareness is related to the subskills of L2 listening.

The correlation between metacognitive awareness and L2 listening (*r* = 0.205, p < 0.01) observed in our study is smaller than those in previous research, for example, 0.44 in Vogely (1995) and Goh and Hu (2013), 0.385 and 0.354 in Zeng and Goh (2015). Two possible reasons can account for the moderate correlation in our study. Firstly, the unfamiliarity of test materials and test structure. According to Zeng and Goh (2015), the stronger correlation in their study derives from the familiarity of test materials and structure and testing environment. As the in-house proficiency test adopted by our study was given to the freshmen, they were ignorant of the test materials and structure before they took the test. Secondly, students’ limited knowledge of metacognitive strategies and their lack of experience in their metacognitive strategy evaluation may result in a moderate correlation between metacognition and test performance, as suggested by Goh and Hu (2013). In our study, the test-takers were taking a comprehensive English course, focusing on improving students’ overall skills of English learning. They had never received any strategy instruction, nor had they ever conducted any evaluation on their own strategy use or instructed to reflect on their mental processes in English learning. It could be possible that some students failed to identify the metacognitive strategies they employed in the test.

### 5.2. To what extent does EFL students’ metacognitive awareness of strategies relate to their listening subskills?

Research question 2 of the current research is concerned with the relationship between listening metacognitive awareness and EFL students’ mastery of listening subskills. When metacognitive awareness was measured as a whole, it was correlated with three of the four subskills, namely, *extracting detailed information*, *making inferences*, and *recognizing main ideas*, respectively. Although correlations do not mean causation, they suggest that metacognitive awareness could be a reliable indicator of students’ listening subskills, but not an independent measure of students’ mastery of listening subskills. This finding suggests that metacognitive awareness operates similarly in the development of these three listening subskills, whether they are high-order subskills (such as *making inferences*) or low-order subskills (such as *detail extraction*). This may pertain to the non-linear progression of comprehension from the lower to the higher processing levels (Field, 2013; Harding et al., 2015). Different levels of listening subskills may be operating simultaneously. When one level fails to work, it may be compensated by another; or when both high- and low- levels break down concurrently, miscomprehension happens (Harding et al., 2015). Significant positive correlations are found between metacognitive factors from the MALQ and all of the listening subskills with *understanding words and syntactic structures* being the only exception. The weak correlation between it and metacognitive awareness might have to do with the methodological issues. In particular, there were only three items tapping into this subskills in the listening test. Using three items meets the minimum requirement of the CDA, but it constitutes a major source of error, thereby weakening the correlations between this subskills and metacognitive awareness (Min et al., 2022b). From a psychological perspective, it’s more difficult to measure and infer learners’ listening vocabulary knowledge from listening tests (Min et al., 2022b) as the parsing of words involves cognitive processing of L2 speech (Field, 2008), which adds to the difficulty of disentangling learners’ vocabulary knowledge from their listening performance unless the test directly taps into the listening vocabulary size of the test participants (McLean et al., 2015). It’s not surprising that test takers’ metacognitive awareness of the listening process explained very small variance in the listening subskills of understanding words and syntactic structures.

In order to further explore the relationship between metacognition and mastery of listening subskills, we examined the correlations between the six components of metacognitive awareness and the four listening subskills, respectively. Overall, all the metacognitive factors were correlated with the mastery of listening subskills. However, the degree of correlation varied when individual factors and subskills were considered.

First of all, the high-order listening subskills, *making inference* and *recognizing main ideas*, produce the strongest correlation with metacognitive factors of *problem-solving, directed attention*, and *planning and evaluating*, respectively; while the low-order listening subskills, *detail extraction and wording understanding*, have the strongest correlation with metacognitive factors of *person knowledge* and *mental translation*, respectively. A possible reason for this pattern has to do with the nature of the metacognitive factors. *Problem-solving* and *planning and evaluation*, for example, involve strategies of making and monitoring inferences, listening planning and effort evaluating (Vandergrift et al., 2006), all of which are closely related to high-order processing. On the other hand, *person knowledge* includes difficulty assessment, learners’ confidence and level of anxiety in listening, and *mental translation* represents inefficient approaches to listening comprehension (Vandergrift et al., 2006). Therefore, the test takers adopting low-ordered subskills, such as *extracting detailed information*, are more likely to have less confidence and more anxiety and resort to *mental translation*, and those who fail to master the low-ordered subskills, such as *understanding words and syntactic structures*, tend to resort to inefficient approaches to listening comprehension by translating key words or utterances into their first language.

Among the five factors, *person knowledge* was the best indicator of the mastery of the listening subskills, especially *detail extraction*, followed by *summarizing main ideas and making inferences*, which suggested that test takers with great confidence and less anxiety have a better mastery of detail extraction, main-idea summarizing and making inferences. This is not surprising given that confidence and anxiety have close relationship with learners’ approach to learning (e.g., Wenden, 1991; Yang, 1999; Zimmerman and Schunk, 2001). Subskill-level statistics, however, displayed an inconsistency regarding the mastery status of the four listening subskills. *Summarizing main ideas* exhibits the most significant mastery status, followed by *understanding words and syntactic structures, extracting detailed information* and finally, *making inferences*. This is reasonable because item difficulty is affected by a variety of factors, including “the setting, test rubrics, input, expected response, and the relationship between input and response” (Min et al., 2022a, p. 18).

*Metal translation* in this study, on the other hand, is negatively correlated with all of the listening subskills, which suggests that listeners with low mastery of listening subskills tend to translate words or entire utterance into their first language. According to previous studies (Eastman, 1991; Goh, 1998; Vandergrift, 2003b), *mental translation* is a metacognitive strategy listeners with low language proficiency feel compelled to use (Vandergrift et al., 2006). It occurs to most learners in the development process of their listening capacity if they receive bottom-up language instructions and build up their understanding of a passage by using individual words and other basic elements of language (Goh and Hu, 2013). Therefore, it’s not unusual to find that test takers in this study resort to mental translation more when they’ve encountered difficulties in extracting detailed information, summarizing main ideas and making inferences, even when they try to understand words or syntactic structures. To become skilled listeners, they must decrease their reliance on interpreting important words or full statements word for word and develop their ability in automatic word recognition and interpretations. As time goes by, they will be able to process text and meaning simultaneously and quickly (Hulstijn, 2003; Segalowitz, 2003).

## 6. Conclusion

This study is a useful complement of resent research on the mental processes of EFL listening by exploring the contribution of Chinese learners’ metacognitive awareness to their listening performance and to listening subskills identified by cognitive diagnostic approaches. It provides further evidence for the impact of metacognitive awareness on listening test performance, suggesting that metacognition has positive relationship with the development of L2 listening proficiency. Moreover, it sheds new light on the role of metacognition in L2 listening development by finer-grained analyses of the relationships between individual metacognitive factors and listening subskills. In addition, it pushes the research agenda further by employing CDA to measure subskills of L2 listening, providing methodological implications for future research.

However, some limitations should be addressed. Firstly, we didn’t examine intra-individual differences in EFL students’ metacognitive awareness at different time points of the listening process. Future research is warranted to involve think-aloud, interviews or diaries to investigate the intrapersonal variations of metacognitive awareness throughout the process of EFL listening. Secondly, no consideration has been given to test-specific mechanisms of listening test when adopting CDA in this study. We hereby recommend that future research would involve test-specific facets as they would be a significant part of variance in listening assessment.

## Data availability statement

The raw data supporting the conclusions of this article will be made available by the authors, without undue reservation.

## Author contributions

YF responsible for the design, data collection, and the first draft and the final revision of the manuscript. SM organized the data base and performed the data analysis. MW revised the draft of the manuscript critically. SZ and XP responsible for design and data collection. All authors played a role in manuscript revision, read, and approved for publication of the submitted version and agreed to be accountable for all aspects of the work.

## Appendix

**APPENDIX TABLE 1:** Metacognitive awareness listening questionnaire (MALQ).

Type scale	Strategy or belief/perception	
Planning-evaluation	1. Before I start to listen, I have a plan in my head for how I am going to listen.	1 2 3 4 5 6
Directed attention	2. I focus harder on the text when I have trouble understanding.	1 2 3 4 5 6
Person knowledge	3. I find that listening in English is more difficult than reading, speaking, or writing in English.	1 2 3 4 5 6
Mental translation	4. I translate in my head as I listen.	1 2 3 4 5 6
Problem-solving	5. I use the words I understand to guess the meaning of the words I don’t understand.	1 2 3 4 5 6
Directed attention	6. When my mind wanders, I recover my concentration right away.	1 2 3 4 5 6
Problem-solving	7. As I listen, I compare what I understand with what I know about the topic.	1 2 3 4 5 6
Person knowledge	8. I feel that listening comprehension in English is a challenge for me.	1 2 3 4 5 6
Problem-solving	9. I use my experience and knowledge to help me understand.	1 2 3 4 5 6
Planning/evaluation	10. Before listening, I think of similar texts that I may have listened to.	1 2 3 4 5 6
Mental translation	11. I translate key words as I listen.	1 2 3 4 5 6
Directed attention	12. I try to get back on track when I lose concentration.	1 2 3 4 5 6
Problem-solving	13. As I listen, I quickly adjust my interpretation if I realize that it is not correct.	1 2 3 4 5 6
Planning/evaluation	14. After listening, I think back to how I listened, and about what i might do differently next time.	1 2 3 4 5 6
Person knowledge	15. I don’t feel nervous when I listen to English.	1 2 3 4 5 6
Directed attention	16. When I have difficulty understanding what I hear, I give up and stop listening.	1 2 3 4 5 6
Problem-solving	17. I use the general idea of the text to help me guess the meaning of the words that I don’t understand.	1 2 3 4 5 6
Mental translation	18. I translate word by word, as I listen.	1 2 3 4 5 6
Problem-solving	19. When I guess the meaning of a word, I think back to everything else that I have heard, to see if my guess makes sense.	1 2 3 4 5 6
Planning/evaluation	20. As I listen, i periodically ask myself if I am satisfied with my level of comprehension.	1 2 3 4 5 6
Planning/evaluation	21. I have a goal in mind as I listen.	1 2 3 4 5 6

This questionnaire is from the metacognitive awareness listening questionnaire: development and validation (Vandergrift et al., 2006, p. 462) with two modifications made to item 8 and item 15 to make the MALQ more applicable for Chinese participants.
